# Evaluation of myocardial fibrosis with cardiac magnetic resonance contrast-enhanced t1 mapping in adults patients with aortic stenosis

**DOI:** 10.1186/1532-429X-17-S1-P359

**Published:** 2015-02-03

**Authors:** Oana A Nastase, Mihaela S Amzulescu, Laurianne Boileau, Jean-Louis J Vanoverschelde, Bernhard L Gerber

**Affiliations:** 1 Cardiology, Institute of Cardiovascular Disease "PROF.DR.C.C. ILIESCU", Bucharest, Romania; 2 Cardiology, Division of Cardiovascular Diseases Cliniques Universitaires St Luc., Bruxelles, Belgium

## Background

Development of diffuse myocardial fibrosis in aortic stenosis (AS) has been associated with an adverse prognosis. Measurement of extracellular volume (ECV) from T1 mapping contrast magnetic resonance (CMR), has been recently validated as a non-invasive method for assessment of such diffuse myocardial fibrosis.Our objective was to evaluate the functional consequences of diffuse myocardial fibrosis, measured by T1-ECV in patients with AS, on systolic and diastolic left ventricular function by transthoracic echocardiography (TEE) and CMR.

## Methods

We prospectively studied 27 consecutive patients with moderate or severe aortic stenosis (67±11 years, 19 men) using 2D TEE and CMR. Exclusion criteria were more than moderate valvulopathy, other than AS, LV wall motion abnormalities or documented myocardial infarct. Indexed LV end-diastolic (LVEDV), end-systolic volumes (LVESV), ejection fraction (LVEF) and mass (LVM) were measured from cine-SSFP images using Simpsons method, and left atrial volume (LAV) was calculated using biplane area-length method. ECV was calculated from T1 measurements using conventional 18 Hb 3-3-5 MOLLI of blood and myocardium before and 15-25 min post bolus injection of Gadolinium, by means of the formula: ECV=(1-hematocrit)(1/T1my.post-1/T1my.pre)/(1/T1blood.post-1/T1blood.pre). The presence of scar was assessed visually on late Gadolinium enhancement (LGE) images.

## Results

25 patients had severe AS, 2 moderate AS. By TEE average peak gradient, mean gradient and surface were 83±27 mmHg, 45 ±6 mmHg and 0.74 ±0.2 cm2 respectively. Average E, A, e', ratio E/A and E/e' were 0.74±0.29 cm/s, 0.86±0.2 cm/s, 0.04±0.01 cm/s, E/A=0.87±0.29 and 16.2 ± 5.9 respectively. By CMR average LVEDVi, LVESVi, LVEF, LAMi and LAVi were 78±22ml/m2, 54±28ml/m2, 64±8%, 95±27 g/m2 and 54 ±19 ml/m2 respectively. Average ECV was 29 ±4%. Small spots of LGE were present in 5 (29%) pts. No patient had infarct like LGE. ECV correlated inversely with LVEF (r=-0.60, p<0.0009), and directly LVEDi (r=0.46, p=0.01), LVESi (r=0.62, p=0.0005) and LAVi (r=0.58, p<0.001). Also the ECV correlated with log-BNP values (r=0.59, p<0.004) but not with the NYHA functional classification. ECV was not significantly associated with LV mass, mass/volume ratio, AS gradient, AS aria and the E/A, E/e' ratio.

## Conclusions

High amounts of interstial fibrosis identified by T1 CMR are associated with worse systolic and diastolic function, but do not correlate with the severity of AS itself. This suggests that ECV could be an interesting parameter to detect maladaptive remodeling in AS prior to development of symptoms.

## Funding

Romanian Society of Cardiology Scholarship.

